# Enzyme-catalysed mineralisation experiment study to solidify desert sands

**DOI:** 10.1038/s41598-020-67566-6

**Published:** 2020-06-30

**Authors:** Linchang Miao, Linyu Wu, Xiaohao Sun

**Affiliations:** Institute of Geotechnical Engineering, Southeast University, Nanjing, Jiangsu China

**Keywords:** Biogeochemistry, Environmental biotechnology, Environmental impact

## Abstract

Sandstorms are meteorological phenomena common in arid and semi-arid regions and have been recognized severe natural disasters worldwide. The key problem is how to control and mitigate sandstorm natural disasters. This research aims to mitigate their development by improving surface stability and soil water retention properties through soil mineralization. The enzymatic induced carbonate precipitation (EICP) is proposed to solidify desert sands and form a hard crust layer on the surface of desert sands. In contrast to micro-induced carbonate precipitation commonly used at room temperatures, EICP had high production efficiency and productivity at a broader temperature range (10–70 °C ±) and significantly improves material water retention properties, which was more suitable to desert environment. Results demonstrate that the enzyme-catalysed mineralisation method can be better resistance to high winds as the number of spraying times increased.

## Introduction

Sandstorm is a severe worldwide natural disaster because of land degradation in arid, semi-arid areas. Globally degraded/desertified land area makes up 6.1 × 10^7^ km^2^, covering nearly 41% of Earth’s land surface and affecting approximately 38% of the world’s population^1,2^. Desertification endangers ecosystem function and influences economic development and stability in arid and semi-arid areas^3–5^. The sandstorm occurs when a front or other strong wind system blows loose sand and dirt from a dry surface^6^. In the north of China, sand and dust from East/Central Asia is frequently transported by sweeping sandstorms over long distances on the scale of thousands of kilometers^7^, even arriving to Japan and Korea across the sea, spreading into the Pacific Ocean, North America, Greenland, and the European Alps^8^. In May 2007, a strong sand and dust storm (SDS) event in Taklimakan Desert, the Xinjiang Uygur Autonomous Region of China, traveled more than in one full circuit around the globe in about 13 days^9^.

Controlling land degradation and mitigating sandstorm remain global challenges today^5^. According the different mechanisms of controlling land degradation, Grainger^10^ summarized the three prevailing methods for combating desertification: engineering, vegetation, and chemical methods. Engineering methods cost a lot of human and material resources. Some researchers, such as Deléglise et al.^11^, Liu et al.^12^, Verdoodt et al.^13^, Witt et al.^14^, think that grazing enclosure is an effective, simple, and direct method to control desertification in grasslands and restore vegetation. However, some studies in grasslands have shown that not all plant communities exhibit the same classic vegetation change patterns and regional differences and the influence of vegetation and soil properties for grazing enclosures have to be studied^12,15–17^. In addition, engineering and vegetation methods cannot be moved and reset according to the surface form and condition of sand accumulation in the field, therefore the function of engineering and vegetation methods for controlling desert sand will be reduction and failure over time.

In recent decade, the microbially-induced calcite precipitation (MICP) method has emerged as an alternative method for improving soil properties^18–24^. MICP is a biogeochemical process which essentially promotes metal ions to bind with acid radical ions to form calcium carbonate minerals^25,26^. The hydrolysis of urea by introduced urease-producing bacteria (e.g., *Sporosarcina pasteurii *(*S. pasteurii*) and *Bacillus megaterium *(*B. megaterium*)) is one of the most popular methods to induce carbonate precipitation^27^. It is widely thought that carbonate precipitation has significant potential as an important method of biomineralization^28,29^. But MICP are very complex and time consuming processes that frequently require special temperature environments. However, EICP has high productivity of calcium carbonate minerals at broader temperatures (ranging from 10 to 70 °C), which was more suitable to desert environment^30^. EICP involves mixing the soil with urea, calcium chloride (CaCl_2_), and urease enzyme^31–34^.

To improve the options for mitigating sandstorm disasters, this paper explored EICP for solidifying desert sands. Experiments to solidify desert sand were conducted in the laboratory with an enzyme solution followed by a urea–calcium acetate solution for different solidification models. The objective of EICP desert sand solidification is to form a hard crust layer on the sand surface to combat wind erosion. Curing effects on solidified desert sand by EICP were evaluated by wind tunnel testing, drying–wetting cycle testing, and water retention feature testing. The effectiveness of EICP, in particular on sand water retention, wind resistance, and environment stability were closely examined. Simultaneously, EICP desert sand solidification is an environmental friend method.

## Results

### Experimental materials

**Figure 1:**
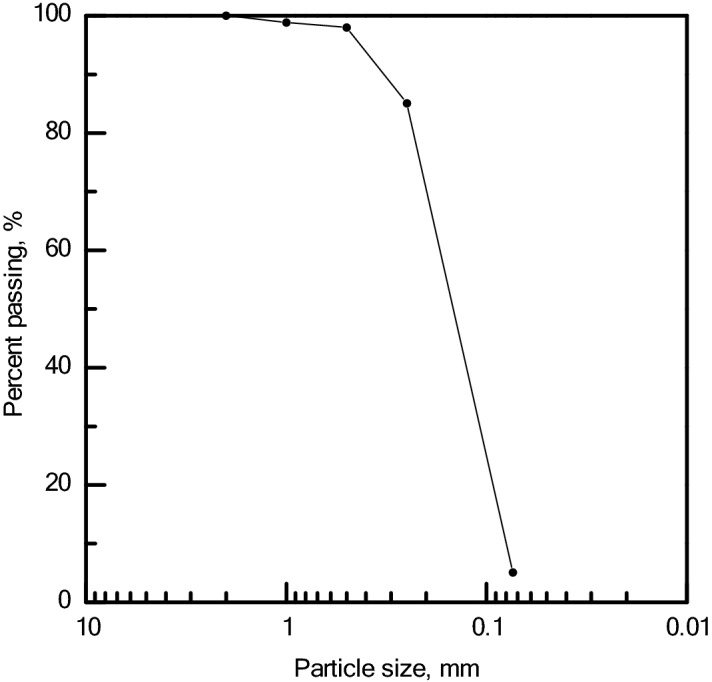
Particle size distribution of Ningxia desert sand.

Tests reported in this paper were performed on desert sand collected from the Tengger Desert in China’s Ningxia Hui Autonomous Region, which is the fourth largest desert in China. Desert sand is fine sand whose particle size distribution is shown in Fig. 1. The physical and chemical properties of Tengger Desert desert sand are listed in the Table 1. Table 2 refers to its mineral components.

**Table 1:** Physical and chemical properties of Desert sand of Ningxia.

Name	Specific gravity	Water content (%)	Stacking density (kg m^−3^)	pH value
Desert sand	2.65	0.5%	1,440.3	8.39

Specific gravity means the ratio of the mass of the soil particles dried at 105–110 °C to the mass of pure water at 4 °C.

**Table 2:** Mineral components of Desert sand of Ningxia.

Name	SiO_2_	Al_2_O_3_	CaO	Fe_2_O_3_	MgO	K_2_O	Na_2_O
%	69.8	12.5	2.67	2.98	2.16	2.78	1.17

### Soil mineralization of EICP

**Figure 2:**
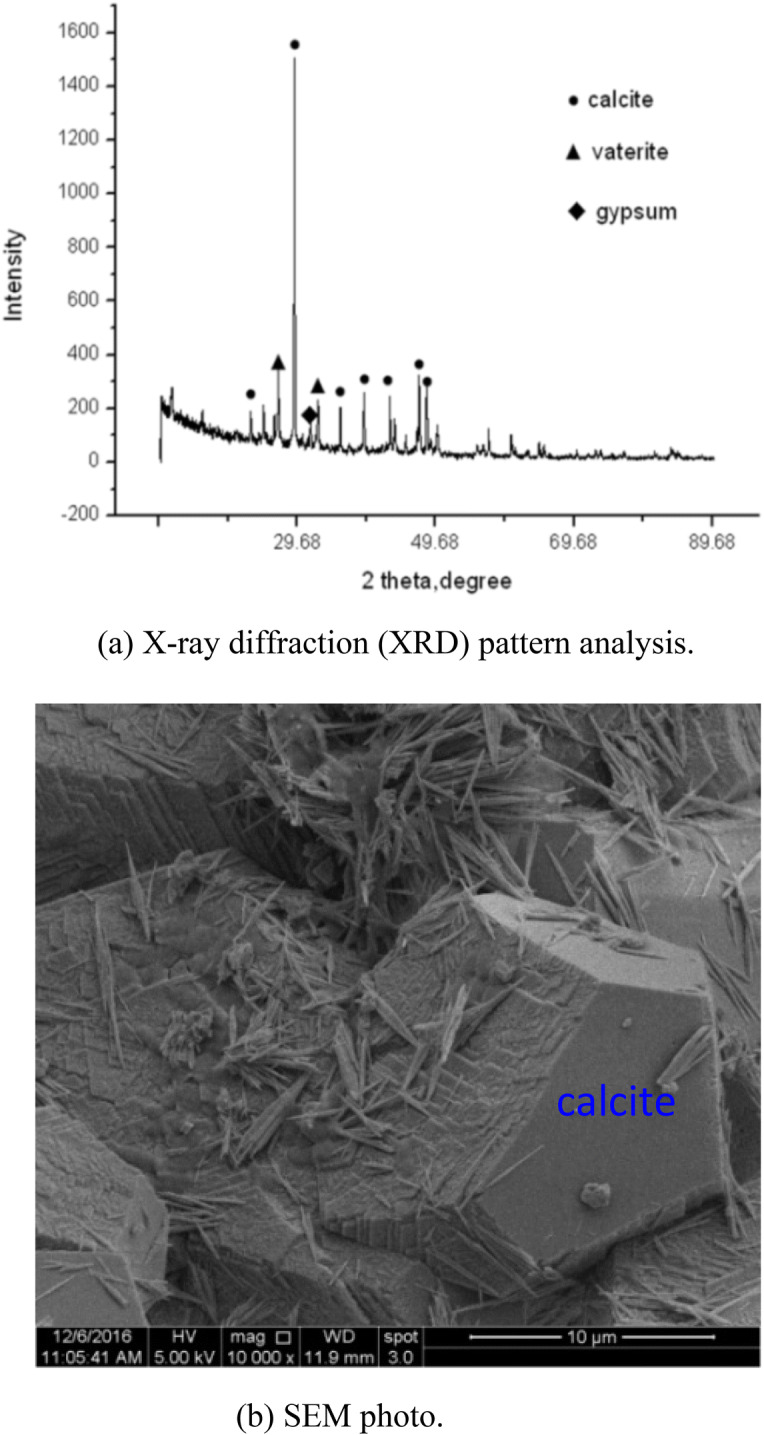
X-ray diffraction (XRD) pattern analysis and scanning electron microscope (SEM) solidification desert sand of EICP.

X-ray diffraction (XRD) pattern and scanning electron microscope (SEM) analysis confirmed that the calcite precipitation was deposited in the desert sand samples by EICP solidification (Fig. 2). SEM showed that the calcite was deposited between desert sand particles and increased their bonding forces. Calcite was sprayed in 6 g, 14 g, and 25 g increments two, four, and six times for the EICP mixture solution, respectively, and the volume increased as spraying times increased.

### Water retention capacity of EICP solidified desert sands

**Figure 3:**
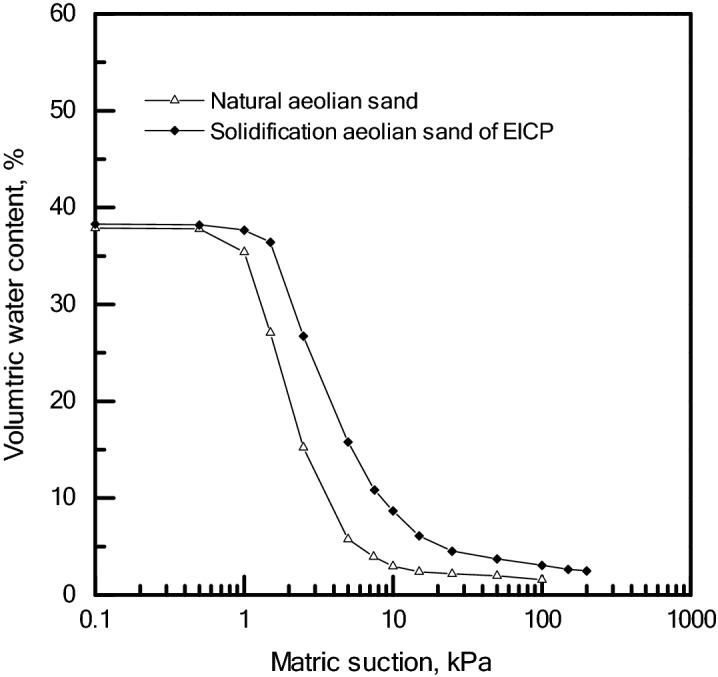
Soil–water retention curves of natural and EICP solidified desert sand.

In deserts, the water retention capacity of natural desert sand is very low and it is easily removed by the wind. The EICP mineralisation method was able to solve the blown sand problem. Figure 3 shows the soil–water retention curve (SWRC) for EICP solidified desert sand. The residual volumetric water content was 6.1% for solidified desert sand and only 2.0% for natural desert sand. The improved water retention capacity of solidified desert sand was confirmed by SEM in Fig. 2. The calcite crystals induced by EICP filled gaps between sand particles and altered the pore structure of natural desert sand. Pores may have been connected, causing water and pore-water to easily evaporate and lose most moisture at the site. The solidified desert sand formed an aggregate of desert sand bonded by calcite crystals, however, and pore structure was different from the natural desert sand. The partial pores solidified desert sand may have been closed or semi-closed. Therefore, the solidified desert sand had improved water retention capacity. Improvements using EICP could facilitate the planting of vegetation to improve the environment in arid and semi-arid areas.

### Effects of EICP solidified desert sands

This study tested solidified desert sand using EICP mineralization technology. The objective was to identify an effective and environmentally responsible technology to control sandstorm disasters. Results demonstrated that the enzyme-catalysed mineralisation method could be used for sandstorm impact reduction and improved water retention capacity in arid and semi-arid areas. (1) EICP soil mineralization can improve the surface stability of desert sand. The calcite added by EICP deposits between particles of desert sand formed an aggregate that alters the sand structure. (2) The soil–water retention curves for solidified desert sand showed that the EICP method enhanced water retention capacity. (3) EICP soil mineralization can be used to consolidate desert sand in desert areas, forming a hard crust layer on the surface that resists sand suspension and fights sandstorm disasters. (4) The calcite precipitation of EICP is stable and long lasting. Wind tunnel and wetting–drying cycle testing of solidified desert sand showed that it could endure strong winds at maximum wind speeds of 29.1 m/s. Mass loss rates for wetting–drying cycle samples of solidified desert sand at 29.1 m/s were 2.31%, 1.87%, and 0.18% after two, four, and six spraying times, respectively. These tests showed that EICP biomineralization technology clearly diminished the harmful impacts of sandstorms. (5) All tests were completed at environment temperatures of 10 °C ±, showing that EICP biomineraliztion technology has a clear obvious advantage over MICP for solidifying desert sand. EICP is environmentally-friendly and can be applied to reduce sandstorm disaster impacts in arid and semi-arid areas.

## Discussions

**Figure 4:**
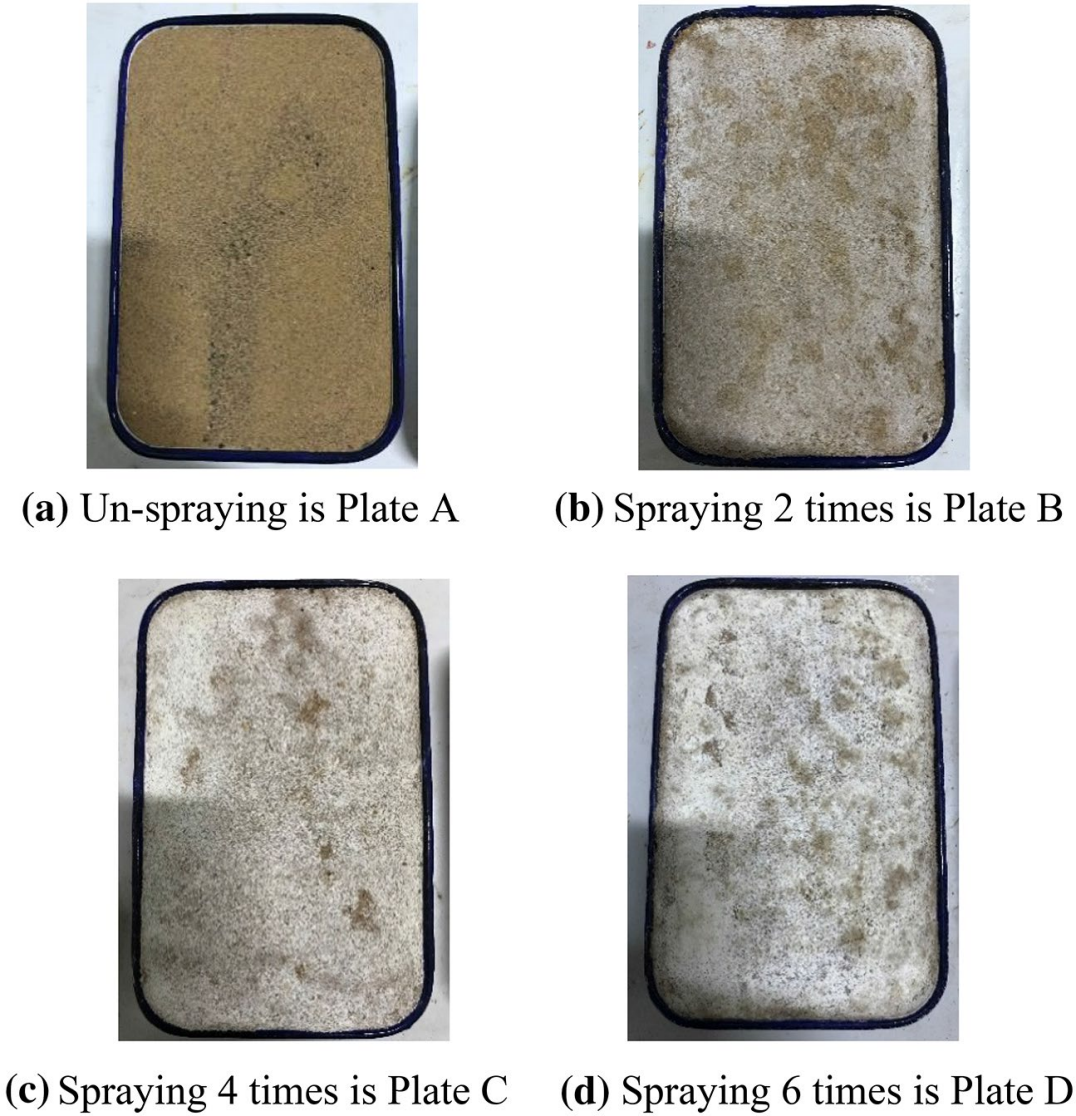
Photos of un-solidification and solidification desert sand of EICP.

**Figure 5:**
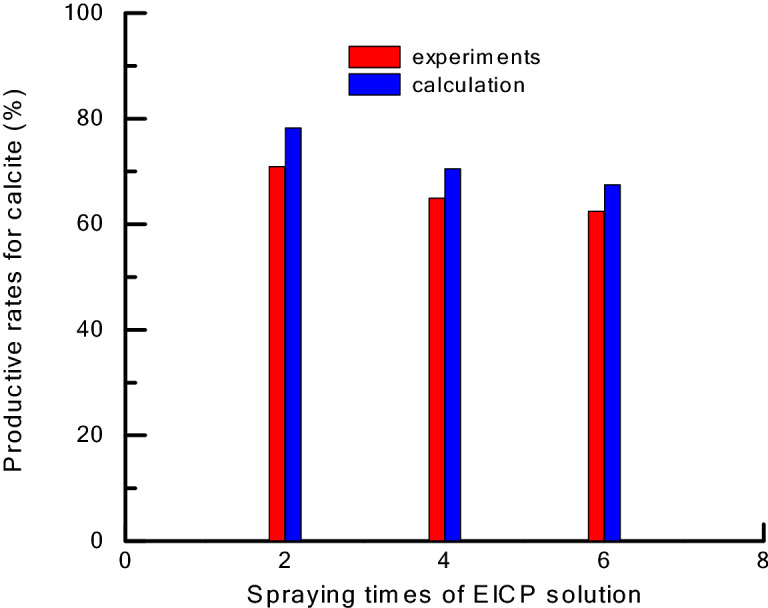
Productive rates of calcite during solidification desert sand of EICP.

Table 3 lists the mass variability for EICP solidified desert sand samples. Increases in mass were due to calcite deposited by EICP consolidation. Calcite deposition solidified the desert sand, forming a hard crust layer on the surface that resisted suspension. Figure 4 contains photos of solidified desert sand samples. It can be seen that the surface color of solidified samples became whiter as spraying time increased. Productive rates of calcite during EICP solidification were measured and calculated in Fig. 5. They were in agreement with the calculated values for calcite during EICP solidification, which decreased as spraying times increased. This was related to the balance between CO_3_^2−^ and Ca^2+^ concentrations and seepage effects of the EICP solution. For these reasons, the surface color of solidified samples appeared more white as spraying time increased in Fig. 4, which indicated that some calcium acetate remained on the surface because the productive rates of calcite did not reach 100% during EICP solidification. This study attempted to increase the calcite efficiency in the next step to improve the EICP consolidation effects for desert sand.

**Table 3:** The mass variations of solidification desert sand samples of EICP.

Spraying times of EICP solution	Initial mass of desert sand (g)	Mass of after spraying (g)	Increment of calcium carbonate (g)
0	2,730	2,730	0
2	2,730	2,736	6
4	2,730	2,744	14
6	2,730	2,755	25

**Figure 6:**
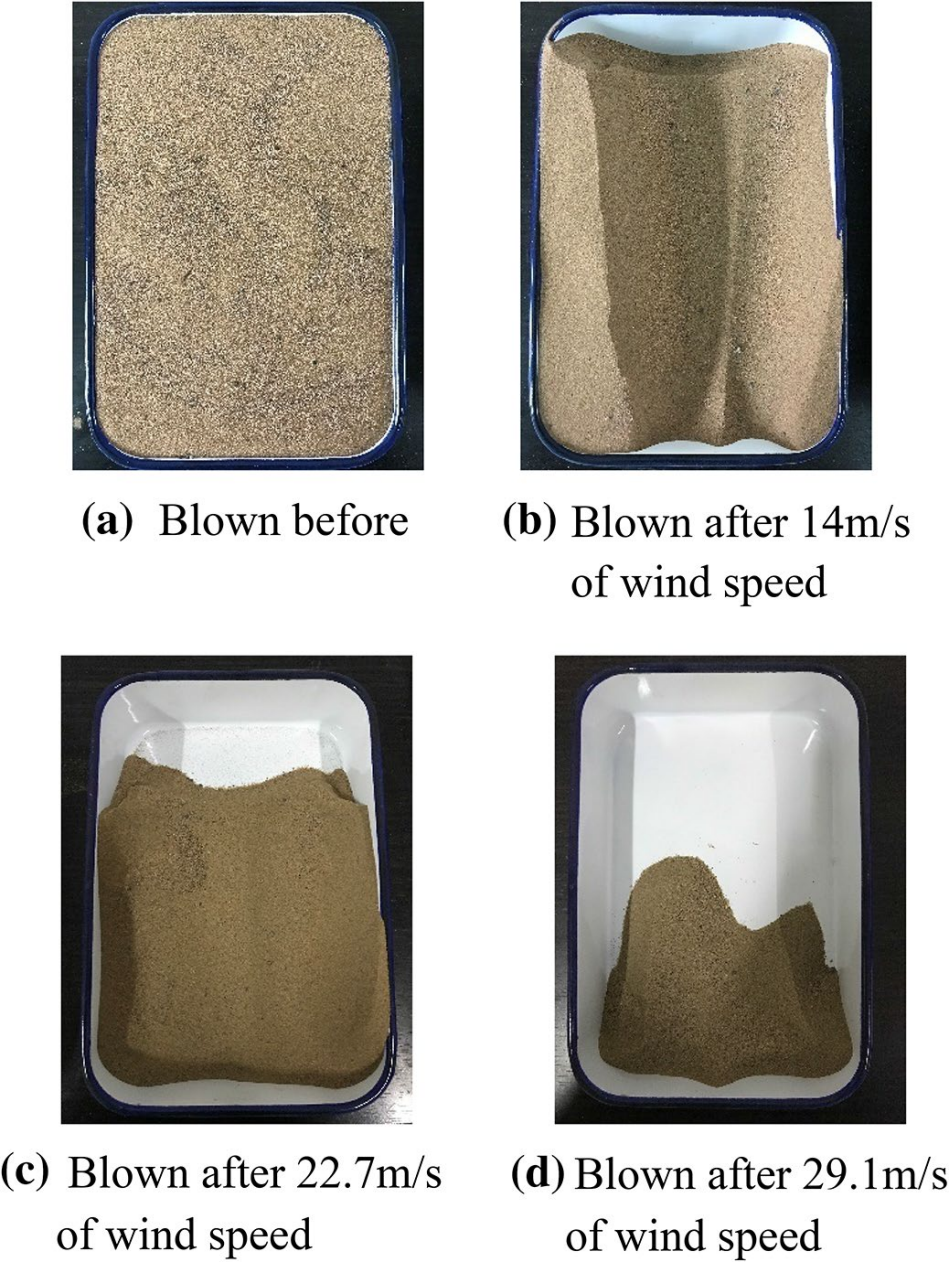
Photos of the natural desert sand blown by different strong winds in 1 min.

Table 4 shows the mass variability of natural /solidified desert sand samples after wind tunnel testing. The loss of mass by natural desert sand was large and it was clearly blown upward during wind tunnel tests. The saltation distance and suspension height for natural desert sand were measured when the wind speed reached 7.0 m/s: saltation distance was about 50–60 cm and the suspension height was about 40–45 cm. According to wind tunnel test data in Table 4, the blown mass loss rates for natural desert sand were 36.4%, 62.7%, and 80.0% at wind speeds of 14.0 m/s, 22.7 m/s, and 29.1 m/s, respectively. These tests aided the interpretation of sandstorm disasters because high amounts of desert sand were removed by strong winds in desert areas (Fig. 6).

**Table 4:** The mass variations of solidified desert sand samples of EICP after blowing at different wind speeds during the wind tunnel tests.

Spraying times	Initial mass (g)	Wind speed: 14.0 m/s		Wind speed: 22.7 m/s		Wind speed: 29.1 m/s	
		Mass blown (g)	Mass changes (g)	Mass blown (g)	Mass changes (g)	Mass blown (g)	Mass changes (g)
0	2,730	1,736	− 994	1,019	− 1,711	426	− 2,184
2	2,736	2,716	− 20	2,687	− 29	2,626	− 61
4	2,744	2,743	− 1	2,735	− 8	2,691	− 44
6	2,755	2,753	− 1	2,751	− 2	2,749	− 3

**Figure 7:**
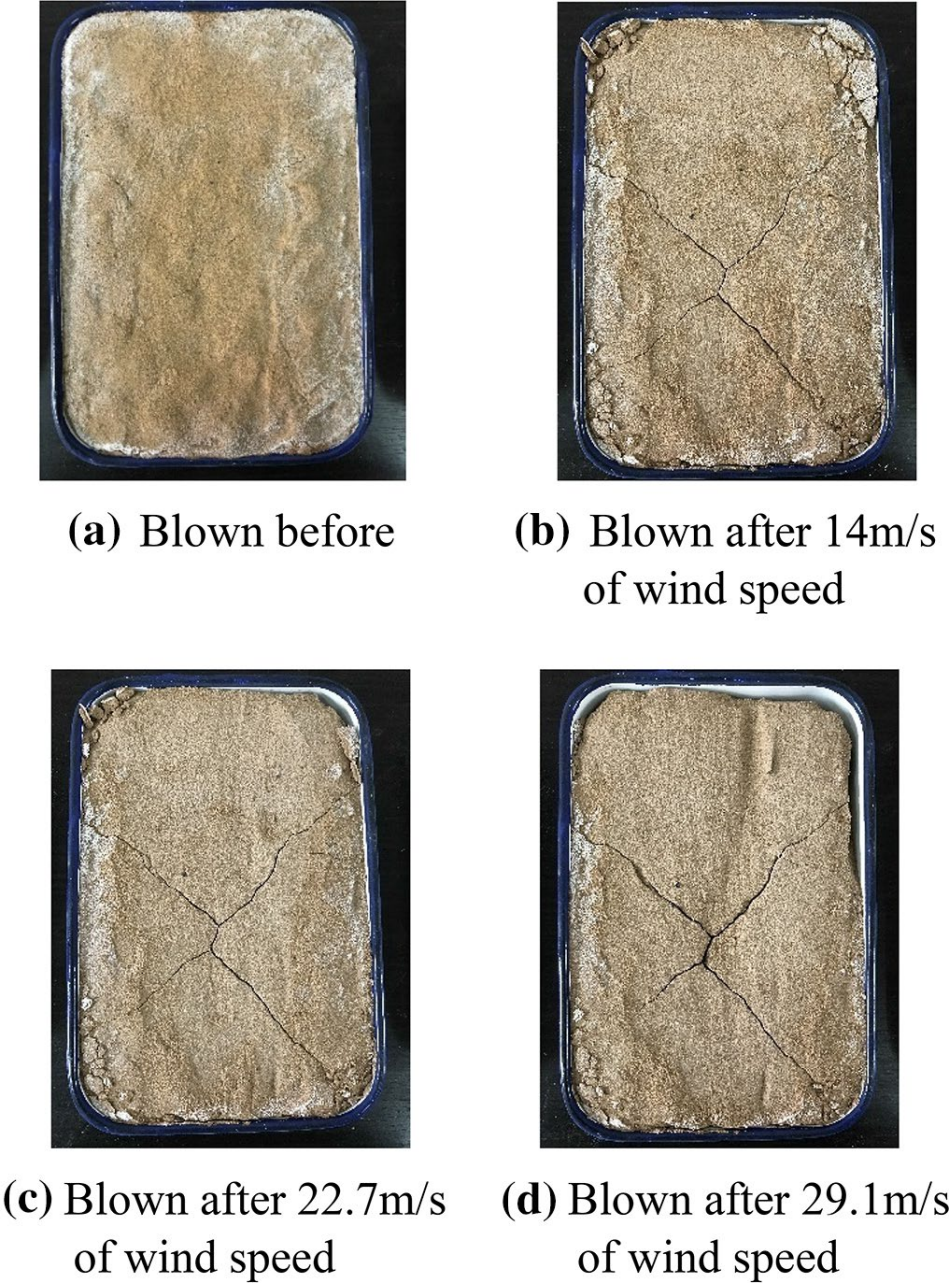
Photos of the solidification aeolian sand blown by different strong winds in 1 min. for spraying 2 times of mixture solution of EICP.

**Figure 8:**
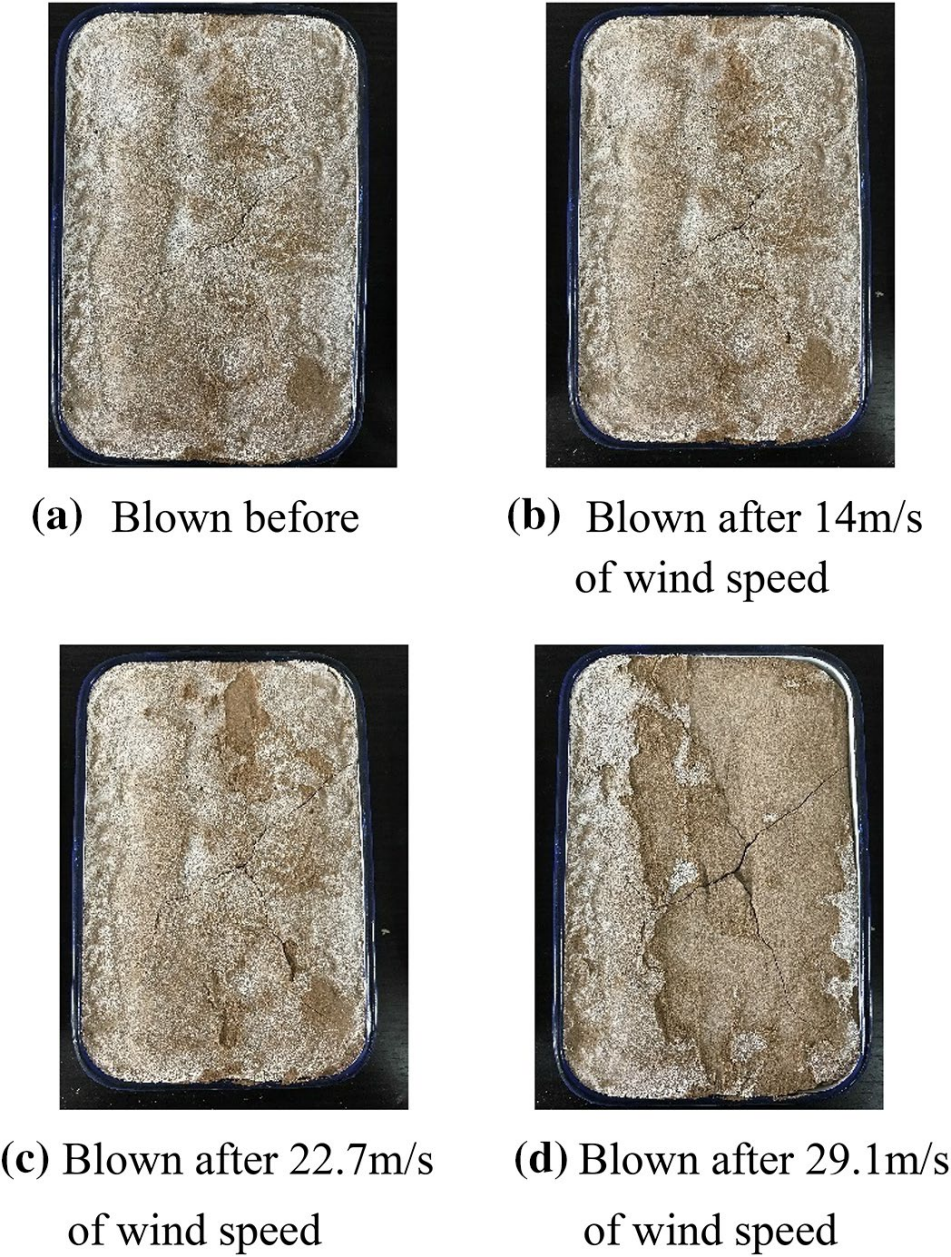
Photos of the solidification aeolian sand blown by different strong winds in 1 min. for spraying 4 times of mixture solution of EICP.

**Figure 9:**
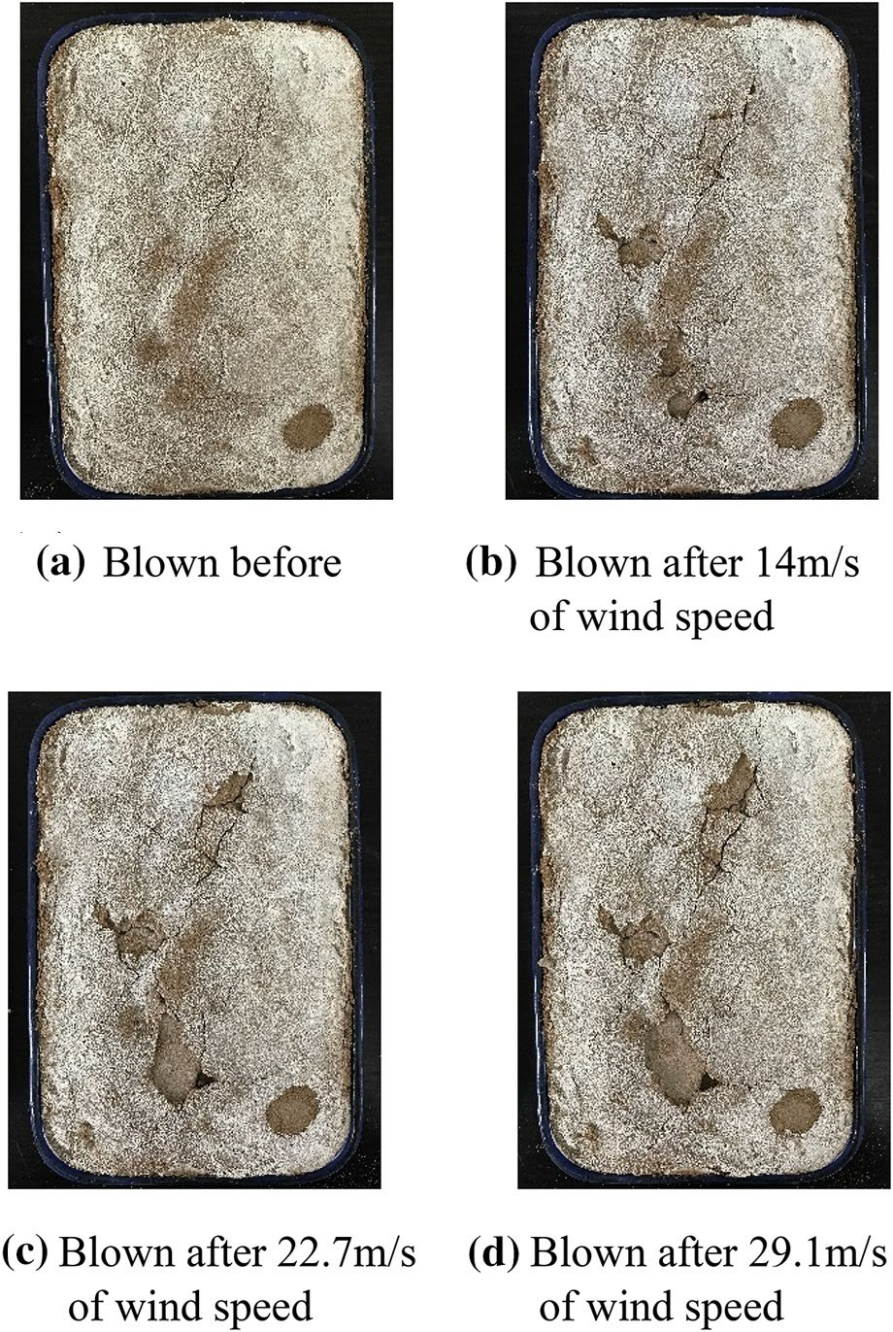
Photos of the solidification aeolian sand blown by different strong winds in 1 min. for spraying 6 times of mixture solution of EICP.

However, Table 4 shows that the solidified desert sand resisted the strong winds and limited sandstorm impacts. During wind tunnel testing, the solidified desert sand samples did not exhibit the suspension and saltation phenomena of natural desert sand, even if winds were low. Higher EICP spraying times also resulted in better solidification effects of desert sand. According to wind tunnel test results in Table 4, the blown mass loss rates of solidified desert sand were less than 3.0% after two spraying times at wind speeds of 14.0 m/s, 22.7 m/s, and 29.1 m/s. Mass loss rates at a wind speed of 29.1 m/s were 2.23%, 1.61%, and 0.11% after two, four, and six spraying times, respectively. Figures 7, 8, and 9 show photos of solidified desert sand under strong winds at different intensities. They demonstrate that EICP was a good method for solidifying desert sand and can be used to limit sandstorm disaster impacts in desert areas. In addition, EICP tests were completed at low environmental temperatures (10 °C ±). This means that EICP can acclimate to broader field conditions and may be more widely applied.

**Figure 10:**
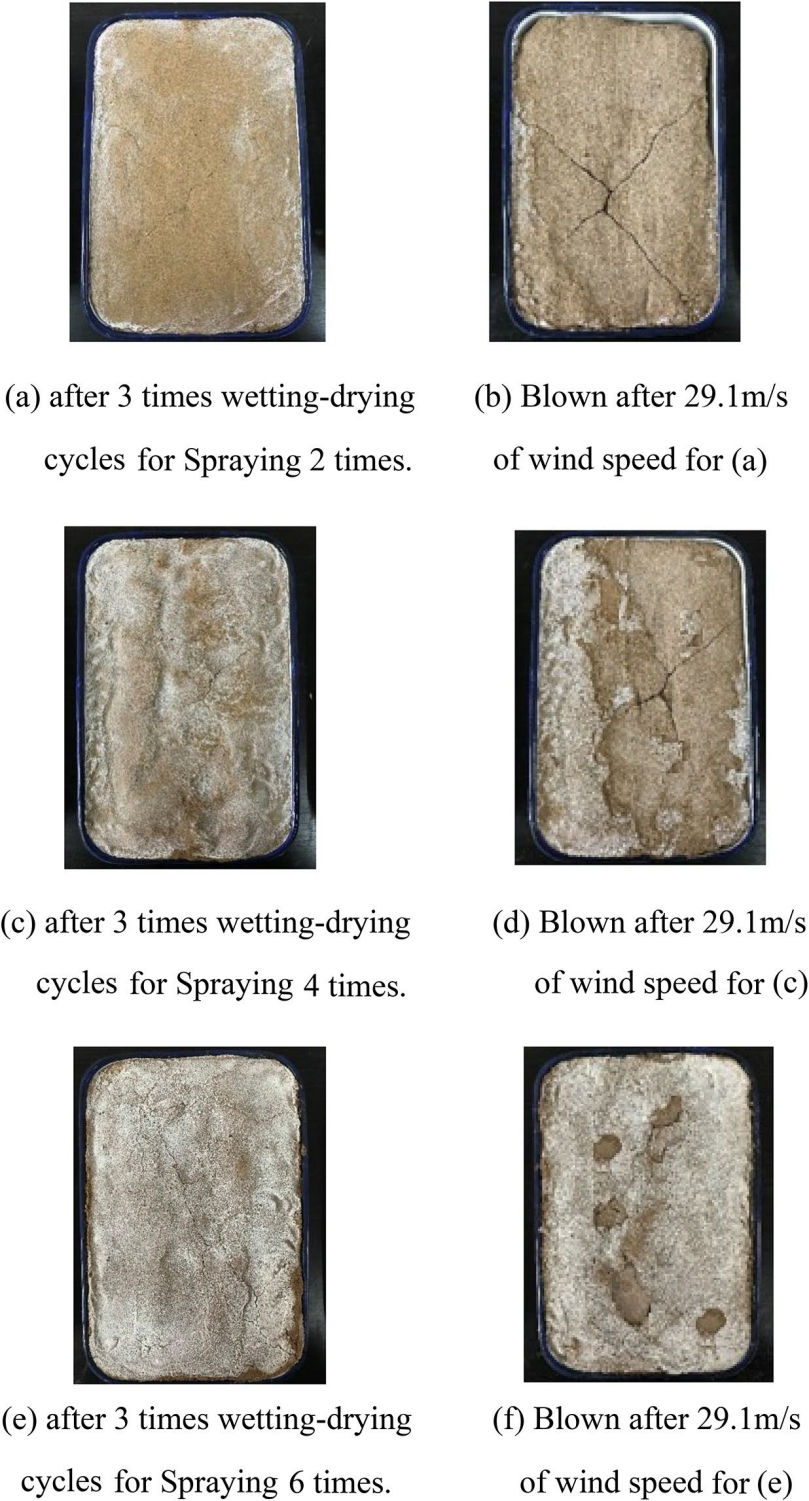
Photos of the solidification aeolian sand blown by strong winds in 1 min. after 3 wetting–drying cycles for different spraying times of mixture solution of EICP.

Table 5 lists the mass variability of solidified desert sand after wetting–drying cycles at different wind speeds. The lack of mass variability in the samples illustrates that the calcite deposition of EICP was not hydrolyzed and was stable over long time periods. The stability and duration of EICP solidification were also demonstrated by wind tunnel tests on wetting–drying cycle samples. Figure 10 shows that wetting–drying cycle samples resisted the impacts of strong winds and did not experience the same saltation and suspension phenomena at a wind speed of 29.1 m/s. During wind tunnel testing, mass losses were less than 3.0% for wetting–drying cycle samples of solidified sand, and the lost components were partial calcium acetate crystals that remained on the surface. The mass loss rates of wetting–drying cycle samples of solidified desert sand at a wind speed of 29.1 m/s were 2.31%, 1.87%, and 0.18% after two, four, and six spraying times, respectively. Therefore, EICP had long-term application potential for consolidating desert sand and limit sandstorm disaster and combat desertification.

**Table 5:** The mass variations of solidified desert sand samples of EICP after blowing at different wind speeds during the wind tunnel tests of wetting–drying cycles.

Spraying times	Initial mass (g)	Wind speed: 14.0 m/s		Wind speed: 22.7 m/s		Wind speed: 29.1 m/s	
		Mass (g)	Mass changes (g)	Mass (g)	Mass changes (g)	Mass (g)	Mass changes (g)
2	2,736	2,715	− 21	2,684	− 31	2,621	− 63
4	2,745	2,744	− 1	2,738	− 6	2,687	− 51
6	2,756	2,756	0	2,755	− 1	2,750	− 5

In addition, the cost of consolidating desert sand of EICP is about the same as the grass square method and the cost estimate is based on EICP test effects in the laboratory and field tests^30^.

## Methods

### Characteristics of reactants and urease enzyme

Urease was extracted from soybeans and purified for use in EICP. It hydrolyses urea (CO(NH_2_)_2_) into carbonate ions (CO_3_^2−^) and ammonium (NH_4_^+^) [Eq. (1)]. The resulting carbonate ions binds with calcium ions (Ca^2+^) supplied by a calcium acetate solution (Ca(CH_3_COO)_2_) to generate calcite precipitate [Eq. (2)].

1 $${\text{CO}}\,\left( {{\text{NH}}_{{2}} } \right)_{{2}} + {\text{ 2H}}_{{2}} {\text{O }} \to {\text{ 2NH}}_{{4}}^{ + } + {\text{ CO}}_{{3}}^{{{2} - }}$$

2 $${\text{CO}}_{{3}}^{{{2} - }} + {\text{ Ca}}^{{{2} + }} \to {\text{ CaCO}}_{{3}} .$$

### Urea and calcium ion concentrations

A urea–Ca (CH_3_COO)_2_ solution was used for EICP. First, urea was added to a solution of urease with 4,000 U/L (1 U corresponds to the amount of enzyme that hydrolyses 1 μmol of urea per minute at pH 7·0 and 25 °C) and over 48 h the urea was hydrolysed by the urease. Then the urea hydrolysed solution was mixed with calcium acetate solution. The calcium ion concentration in calcium acetate was 0.75 mol/L and the urea concentration was 0.75 mol/L in the EICP solution.

### Water retention feature testing

Water retention feature tests were conducted by compression with the soil–water retention curve (SWRC) test on unsaturated soils. The sampling density of SWRC was 1.6 g/cm^3^ for natural and solidified desert sand. The size of all samples for water retention feature testing was 61.8 mm in diameter and 20 mm in height. Solidified desert sand samples were sprayed with 10 mL of the EICP mixture solution. Calcium ion and urea concentrations of the EICP solution were 0.75 mol/L as mentioned above. The natural and solidification desert sand samples were saturated prior to water retention tests. During water retention testing, matrix suction was set to increase in 0.5, 1.0, 1.5, 2.5, 5.0, 7.5, 10.0, 15.0, 50.0, 100.0, 150.0 and 200.0 kPa increments for 24 h and tests were only conducted for drying path.

### Temperatures and pH values

The temperature was 10 °C ± during the EICP reaction and the pH of the solution was 7. Based on the desired engineering application of the enzyme-catalysed mineralization method, the low temperature was controlled during solidification desert sand tests.

### Solidified desert sand

Four plates were prepared to fill desert sand (A, B, C, and D). Plate size was 16 × 24 × 4 cm and each plate contained 2,730.0 g of desert sand from Tengger Desert. The EICP solution was sprayed for various durations onto the desert sand on the plate to solidify it. The urea–Ca (CH_3_COO)_2_ and urease solutions were sprayed in 50 mL amounts each time for a total amount of 100 mL. Plate A was not sprayed with EICP. Plate B was sprayed twice with 48 h in between. Plate C was sprayed four times in 48 h intervals. Plate D was sprayed six times in 48 h intervals.

### Wind tunnel testing for solidified desert sand

To evaluate the solidification effects, wind tunnel and drying–wetting cycle testing was conducted on solidified desert sand. The following procedure for wind testing was followed:

1. Drying phase: The wet solidified samples of desert sand on plates B, C, and D were kiln-dried in an oven for 8 h at 60 °C in the laboratory and then weighed.
2. Wind tunnel testing: Tests were conducted at three different wind speeds, 14.0 m/s, 22.7 m/s, and 29.1 m/s. Plate A contained natural desert sand, and plates B, C, and D contained solidified desert sand sprayed two, four, and six times with EICP solution, respectively. All plates were placed in a wind tunnel and blown for 1 min at different wind speeds: 14.0 m/s, 22.7 m/s and 29.1 m/s. Then the desert sand samples were weighed again.

### Wetting–drying cycle testing for solidified desert sand

The following procedure was used for drying–wetting testing:

1. Wetting phase: The solidified samples of desert sand on plates B, C, and D were weighed and then sprayed with 200 mL of tap water, then retained for 24 h in the laboratory.
2. Drying phase: The wet solidified samples of desert sand on plates B, C, and D were kiln-dried in an oven for 8 h at 60 °C in the laboratory, and then weighed. The dried samples of desert sand on plates B, C, and D were stored in the laboratory for a second day.
3. The above procedure was repeated for three wetting–drying cycles.
4. Wind tunnel testing: The dried solidified samples of desert sand on plates B, C and D were placed in a wind tunnel and blown for 1 min at different wind speeds, 14.0 m/s, 22.7 m/s and 29.1 m/s. Plates B, C, and D of solidified desert sand samples were weighed after each test.
